# Knowledge mapping of severe fever with thrombocytopenia syndrome: a bibliometric analysis

**DOI:** 10.3389/fmicb.2024.1423181

**Published:** 2024-07-30

**Authors:** Huiying Zhang, Leiliang Zhang

**Affiliations:** ^1^Department of Clinical Laboratory Medicine, The First Affiliated Hospital of Shandong First Medical University, Shandong Provincial Qianfoshan Hospital, Jinan, China; ^2^Department of Pathogen Biology, School of Clinical and Basic Medical Sciences, Shandong Academy of Medical Sciences, Shandong First Medical University, Jinan, China

**Keywords:** SFTS, DBV, bibliometric, CiteSpace, VOSviewer

## Abstract

**Background:**

Severe fever with thrombocytopenia syndrome (SFTS), caused by the Dabie bandavirus (DBV), formerly known as the SFTS virus (SFTSV), is characterized by rapid progression, high morbidity, and mortality. This study aims to analyze the current research status, hotspots, and trends of SFTS since 2009 through bibliometrics, focusing on original research and providing valuable references and inspirations for future basic research, prevention and control of SFTS.

**Methods:**

The Web of Science Core Collection (WOSCC) was used to extract global papers on SFTS from 2009 to 2024. VOSviewer and CiteSpace software were also used to process and visualize results.

**Results:**

A total of 760 publications relevant to SFTS were reviewed. Among these publications, the most active country, author, and publication type included China, Liu Wei, and original articles, respectively. Among the institutions, the National Institute of Infectious Diseases emerged as the top publisher. The most frequently used keywords were “China,” “Bunyavirus,” and “person-to-person transmission.” The bibliometric analysis reviewed and summarized the research results in the field of SFTS and demonstrated the research trends in the field. In addition, the study revealed the current research hotspots and predicted the future research frontiers and potential challenges in the field of SFTS, which will provide references for further exploring and investigating the SFTS-related mechanisms and inspire new therapeutic strategies.

**Conclusion:**

Bibliometric visualization provides an overview of research advances, hotspots, and trends regarding SFTS and consolidates existing knowledge. SFTS research is in a phase of rapid development, and the number of annual publications in the field is growing steadily and rapidly. This is laying the groundwork for further research and providing new ideas for clinicians engaged in SFTS-related therapies and researchers working to improve public health. Currently, researchers are focused on elucidating the biology of SFTS, exploring antibodies, delving into pathogenesis, and investigating specific therapies.

## 1 Introduction

Severe fever with thrombocytopenia syndrome (SFTS) is an emerging infectious disease caused by the Dabie bandavirus (DBV), formerly known as the SFTS virus (SFTSV) (Yu et al., 2011). SFTS manifests as a hemorrhagic fever that occurs sporadically and carries a mortality rate ranging from 5 to 40% (Li et al., 2021). For individuals over the age of 40, the incidence of SFTS is significantly higher, and increasing age is strongly associated with worsening SFTS and increased mortality (Ding et al., 2014). The first case of this disease has been reported in the Mainland of China, followed by Japan, South Korea, Vietnam, Taiwan are of China, and many other countries and areas, with serious implications for human health in countries around the world. SFTSV is transmitted to humans through tick bites, mainly from the long-horned blood tick, *Hemaphysalis longicornis, Amblyomma testudinarium*, Japanese hard tick, and *Rhipicephalus microplus*, and human-to-human transmission has also been reported with domestic animals (including goats, dogs, and cows) as potential amplifying hosts (Casel et al., 2021). Age is a primary risk factor for hospitalization and death in patients with SFTS, and the principal clinical signs of severe SFTSV infection include hemorrhagic fever, gastrointestinal symptoms, myalgias, arthralgias, dizziness, chills, and localized lymph node enlargement. The most common laboratory abnormalities are thrombocytopenia (95%), leukopenia (86%), and elevated serum alanine aminotransferase, aspartate aminotransferase, and lactate dehydrogenase levels (Zhang et al., 2013). In severe cases, systemic organ failure occurs, which can be fatal (Kim and Park, 2023). SFTSV has rapidly changed due to evolution, genetic mutations and recombination between different SFTSV strains (Zhan et al., 2017). In addition to increasing human health problems associated with SFTSV, the pathogenesis of SFTSV in humans is not fully understood, and there are still no therapeutic approaches for the virus. Understanding all aspects of host-virus interactions following SFTSV infection is critical, including antiviral status and viral escape mechanisms. Thus, weather conditions, modes of transmission, and the creation of new therapies, such as specific vaccines and drugs, are major areas of upcoming research (Saijo, 2022).

Bibliometrics has been widely used in literature analysis. Through bibliometric analysis, researchers can obtain detailed information, including authors, keywords, journals, countries, institutions, and references, which benefits for better understanding the development of a field and its trends. To elucidate the current state of research and trends in SFTS research, we aimed to assess the knowledge mapping of SFTS through bibliometric analysis. Through the bibliometric analysis of SFTS, researchers can identify the research trends and hot issues in this field, which has an essential guiding role in the research and prevention of SFTS. In addition, bibliometric analysis can help researchers assess the impact of research papers, authors, or journals in SFTS-related fields. The influence and importance of SFTS research can be more objectively measured by the number of citations, citation frequency and other indicators, thus providing strong support for academic evaluation and research fund application. In addition, analyzing the collaborative relationships in the literature can reveal the cooperative networks and patterns among countries, authors and institutions, which is beneficial for establishing suitable research cooperative relationships, finding potential collaborators, and promoting academic exchanges. Analyzing the institutions and journals that publish the most relevant papers in the field of SFTS can provide valuable guidance for researchers to select cooperative institutions and appropriate journals to publish papers. Finally, by tracking the time series changes in literature, knowledge evolution and research progress in SFTS can be deeply understood, which helps researchers inspire their research innovation. SFTS literatures published in the Web of Science Core Collection (WoSCC) since 2009 was analyzed using VOSviewer and CiteSpace analysis software. The visualization charts were drawn, including the number of publications, countries, institutions, issuing journals, prolific authors, and keyword co-occurrence analysis. The history of the development of SFTSV, the current status of the research, the current hotspots of the research and the development trend of the frontiers were displayed to provide a new perspective and valuable reference for the future of the SFTS research and prevention work to provide a new perspective and valuable reference basis.

## 2 Materials and methods

### 2.1 Data collection

**Figure 1:**
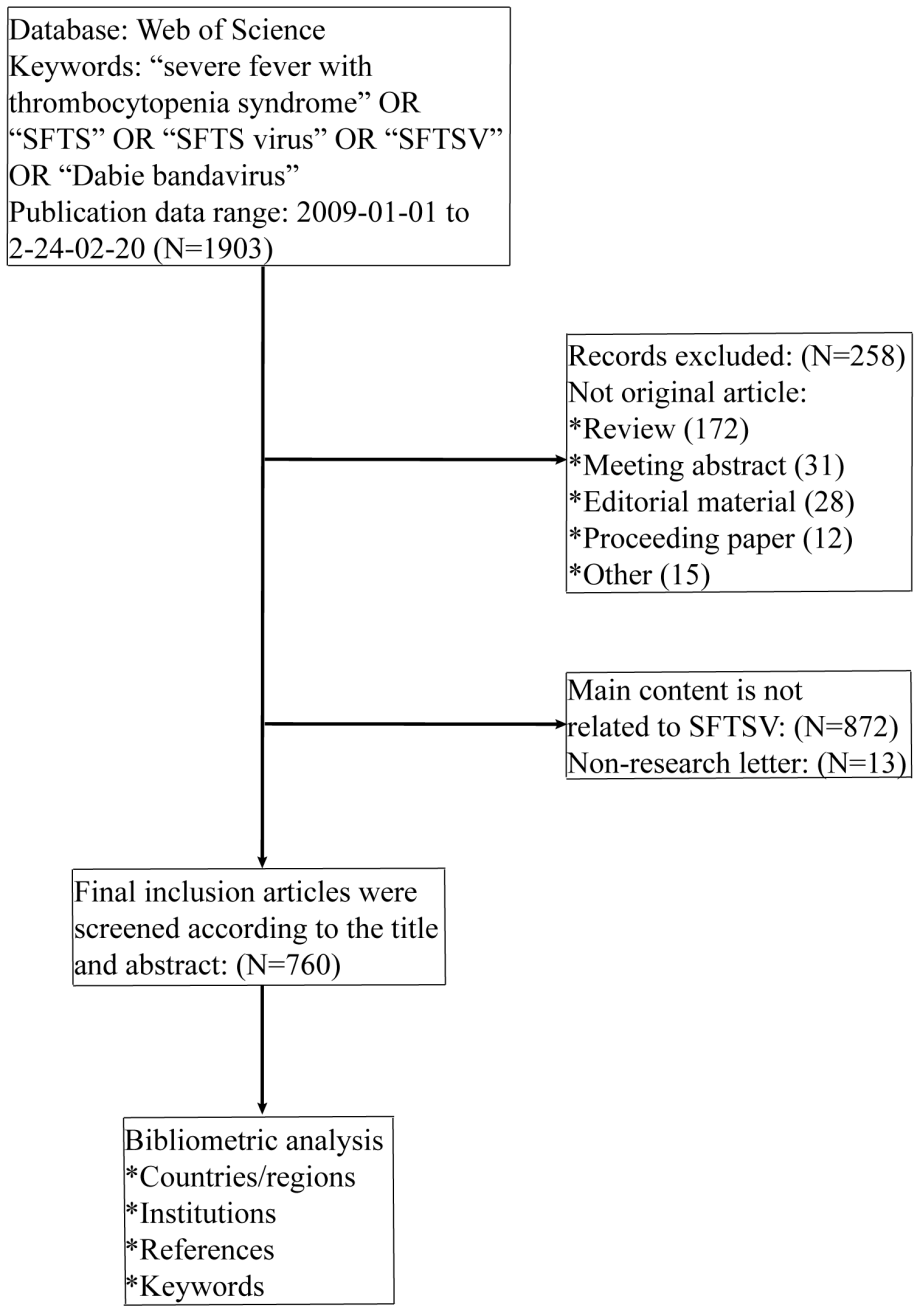
Process of data retrieval.

WoSCC was chosen as the data source for this study, which has been embraced by many researchers as a highly respected database of high-quality digital literature resources and is regarded as the database of choice for bibliometric analyses. We used the following search terms to retrieve literature from the WoSCC database: Topic = (“severe fever with thrombocytopenia syndrome” OR “SFTS” OR “SFTS virus” OR “SFTSV” OR “Dabie bandavirus”), spanning from January 1, 2009 to February 20, 2024. Only original research articles and research letters were included in the file types. The language is “English.” The “severe fever with thrombocytopenia syndrome” and “SFTS,” as subject nouns and acronyms, were used as components of the search terms. The terms “Dabie bandavirus,” “SFTS virus,” and “SFTSV” were used as the pathogenic factor and acronym of SFTS, respectively. Using these keywords in title searches can help researchers more directly and accurately find studies related to SFTS, thereby contributing to a deeper understanding of the characteristics, immunological prevention, pathophysiology, and other aspects of SFTS. To ensure that the data had not been updated, two researchers conducted a comprehensive search and screening of the literature on SFTS within the same day using titles, abstracts, and keywords, reading the full text when necessary, selecting references that showed complete records and citations, and exporting the data retrieved from the Web of Science platform to “TXT” format. The flowchart is shown in Figure 1. No additional ethics committee approval was required as the data for this study were all derived from a public database.

### 2.2 Data analysis and visualization

Co-citation analysis is a bibliometric methodology that focuses on analyzing the relationships between different documents due to being simultaneously cited by the same document or documents. This methodology can help researchers reveal academic papers’ or authors’ interconnectedness and influence. Collections of co-cited papers provide a novel approach to understanding the scientific structure, and analyzing co-citation networks can reveal significant sources of influence and trends in a field of study (Small, 1973). Co-word analysis is a bibliometrics technique mainly used to analyze the co-occurrence of words in the literature. It reveals the relevance and structure of research topics or concepts by identifying keywords that frequently co-occur in different literatures. Co-word analysis allows researchers to identify core concepts and themes in the field of study and understand the connections and dynamics between different themes. Both co-citation analysis and co-word analysis are essential scientific research methods in bibliometrics. They help researchers better understand the dissemination and evolution of scientific knowledge in related fields (Callon et al., 1991).

We built the visualizations using CiteSpace software (version 6.2.4.24) and VOSviewer software (version 1.6.20), which are based on different data analysis methods, so each has its advantages and creates strong synergies. VOSviewer uses probability-based data normalization methods to provide visualization methods such as network view, overlay view, and density view, focusing on showing a more comprehensive network structure, including multiple relationships between nodes, which may result in a dense network. Still, it is easy to draw and operate flexibly (van Eck and Waltman, 2010). This study used VOSviewer to draw a visual knowledge map showing multiple relationships, such as keyword co-occurrence, co-institutions, and co-authors. In contrast, CiteSpace is more challenging to operate. It uses data normalization methods based on set theory to quantify the similarity of data units, generate time zone views, and focus more on displaying critical paths and significant clusters. Therefore, the results are more concise, which helps researchers to understand the development process and trend of the SFTS field and conduct evolutionary analysis. This paper used CiteSpace to draw the cluster diagram of keywords and journals and the dual-map overlap of journals.

In the data pre-processing stage, we used CiteSpace’s data processing utilities to de-duplicate and organize the included literature in WoS to ensure its accuracy. In addition, in the total amount of included literature, we need to carry out a specific process of standardization of the data to ensure its quality and consistency. We create a new “TXT” file following the format of “thesaurus_authors” in the VOSviewer folder and merge synonyms. We implement in “TXT” according to the corresponding format: (1) Merge synonyms. (2) Merge subordinate units into superior units. (3) Merge subordinate territories into the country. We then import it into the “VOSviewer thesaurus file” page in the clustering page of VOSviewer and achieve data reintegration. This data integration method applies to countries, institutions, and references.

## 3 Results

### 3.1 Analysis of publications and citations

**Figure 2:**
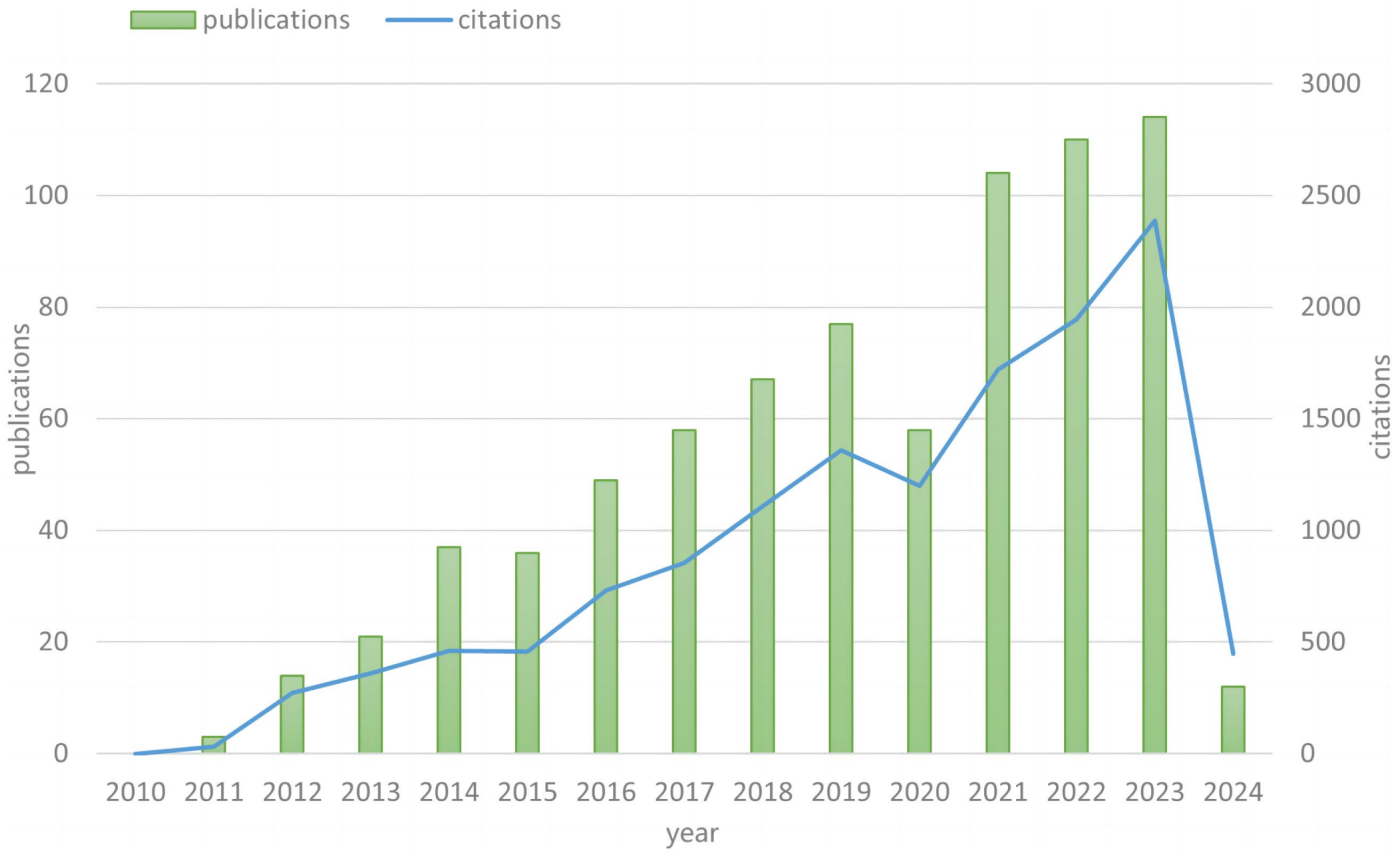
Trends of publications and citations from 2010 to 2024.

The search results of the WOSCC database were processed using CiteSpace, and 760 relevant records were included, including 737 articles (96.97%) and 23 research letters (3.03%). Figure 2 shows the trend of research in the field of SFTS. From 2010 to 2024, the growth trend in the number of papers steadily increases. Especially after 2021, there is a rapid increase in the number of papers published. The annual number of papers published from 2021 to 2023 has stabilized at more than 100, which indicates that this field has received more and more attention from scholars since 2021. The reasons for the sudden increase in the number of publications in the field of SFTS mainly include a combination of factors such as a significant increase in the severity and concern for the disease, a yearly increase in the number of case reports, an emphasis on the need for prevention and control strategies and health education, and an increase in research investment and academic competition.

### 3.2 Analysis of countries/regions

A total of 26 countries and regions published articles on SFTS, of which the top 5 countries with the most publications are shown in Table 1. Chinese scholars have published the most research papers in this field, totaling 447 papers. South Korea and Japan were the second and third most prolific countries, with 164 and 145 papers, respectively. Table 1 also shows that the country with the highest average citations per article is the United States, with 53.26 average citations per article, followed by Germany and China, with 31.20 and 23.30 average citations per article. Total link strength (TLS) measures how tightly nodes are connected. Higher TLS represents more frequent cooperation between that country and other countries. As shown in Table 1, China and the United States are the two most prominent performers in international collaboration.

**Table 1:** The top 5 countries in number of publications on SFTS.

Rank	Country	Publications	Total citations	Average citations	TLS	Centrality
1	China	447	10,415	23.30	106	0.95
2	South Korea	164	3,258	19.87	34	0.42
3	Japan	145	2,912	20.08	35	0.55
4	The United States	84	4,474	53.26	94	0.28
5	Germany	10	312	31.20	11	0.02

**Figure 3:**
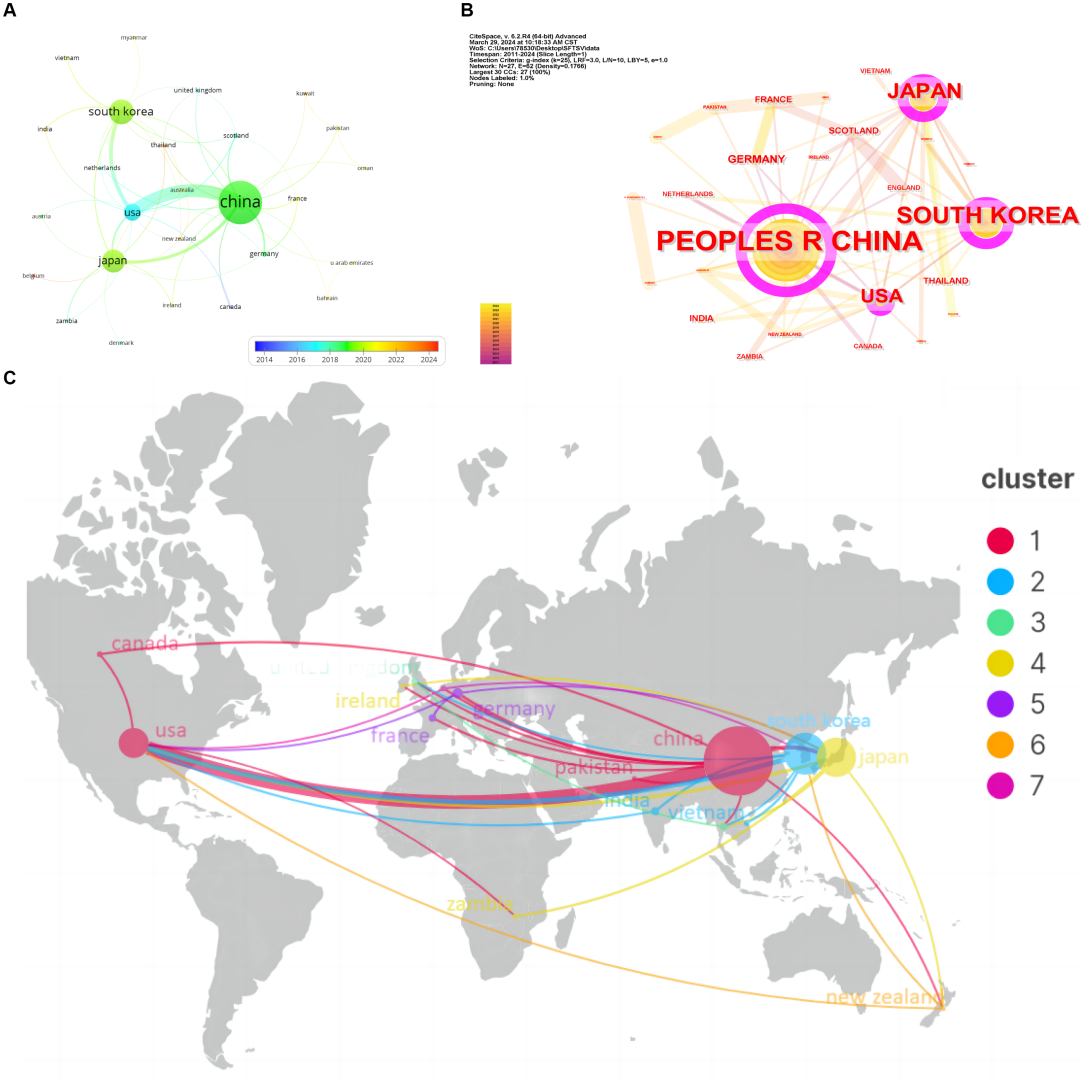
Visualization and analysis of the international collaboration networks in SFTS research. **(A)** Cooperation clustering map of counries. **(B)** Centrality map of countries. **(C)** Co-occurrence of countries by scimago graphica.

Firstly, we visualize the countries with more than or equal to 1 article through VOSviewer, and a total of 26 countries and regions reach the threshold, and the results are shown in Figure 3A. The node size represents the number of articles issued; the node connecting line represents the strength of the association. The thicker the connecting line indicates, the more vital the role of cooperation between two countries; the node color represents the different years of issuance. The bluer the color, the earlier the country publishes an article. The redder the color, the later the country publishes an article. Figure 3B shows that China has the highest centrality (0.95), indicating it has the most collaboration with other countries. The four countries contributing the most to the field of SFTS are China, South Korea, Japan, and the United States, with China having the most collaboration with the United States and Japan. The distribution of issuing countries in this field is very uneven, and the top effect is very significant, with most of the articles being authored by scholars from a few countries.

The cluster in Figure 3C represents the core countries that have published in the field of SFTS, from which it can be seen that China, Japan, South Korea, and the United States are the four countries with the most publications in the field of SFTS. The larger the round nodes in the figure, the larger the number of publications; the node connecting lines represent the strength of the association, and the thicker the connecting lines indicate that the two countries have cooperated more frequently in publishing articles. China, Korea, Japan, and the United States have made core contributions to SFTS.

### 3.3 Analysis of institutions

**Figure 4:**
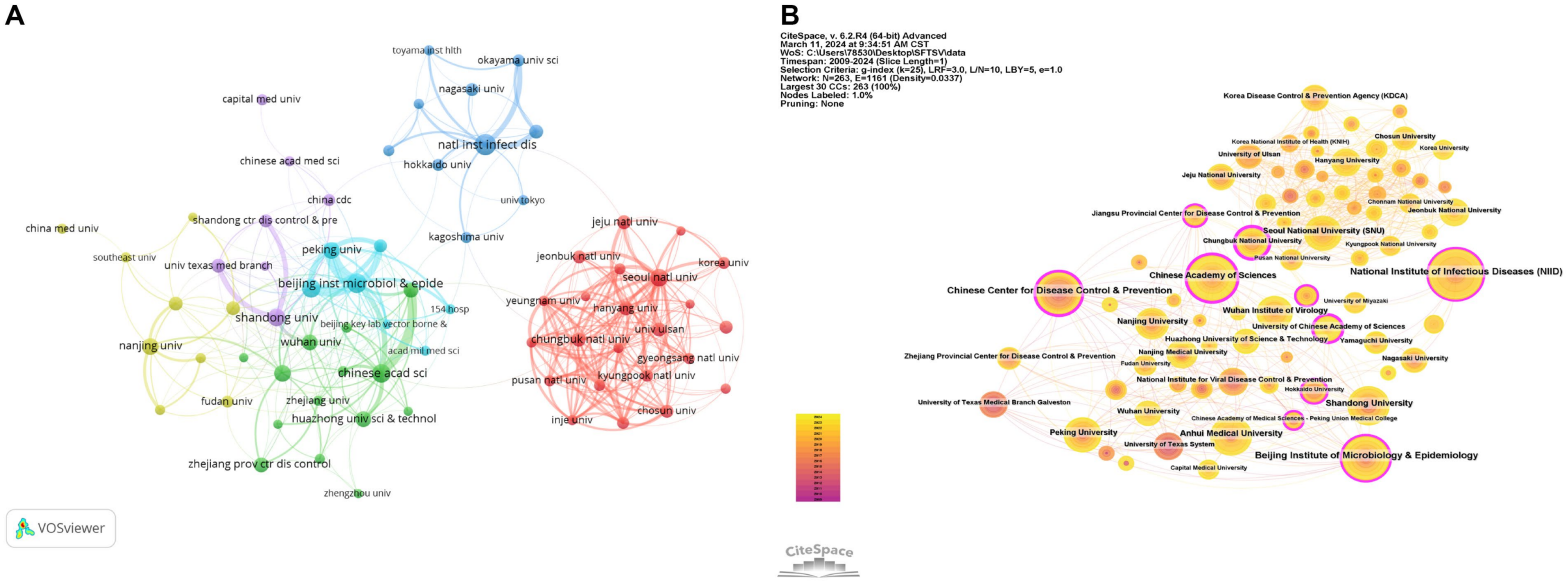
Visualization and analysis of the institutions in SFTS research. **(A)** Cooperation map of 70 institutions with the number of publications no less than 7 times. **(B)** Centrality cooperation map of institutions.

A total of 896 institutions published articles related to SFTS, and the top five institutions with the most publications, as shown in Table 2, were all from China, indicating that China has the largest number of researchers in SFTS-related fields. The National Institute of Infectious Diseases published the highest number of articles (*n* = 80), followed by the Beijing Institute of Microbiology and Epidemiology (*n* = 66) and the Chinese Academy of Sciences (*n* = 58). Setting the VOSviewer parameter to the minimum number of documents for an institution = 7, 70 institutions were obtained. As shown in Figure 4A, the colors symbolize different collaboration clusters, with Japan in blue, South Korea in red, and China in green, purple, yellow and cyan. The vast majority of which are confined to a single country. The connecting line represents the strength of the cooperation association; the thicker the connecting line represents the more cooperation between two institutions to issue documents; the node’s size represents the number of documents issued; the larger the node, the more documents issued. As shown in Figure 4B, CiteSpace is used to construct a visualization diagram of collaborative institutions. The outermost purple circle indicates the betweenness centrality (BC), which indicates the importance of nodes in the network. Larger purple circles indicate larger BC, which indicates the contribution of institutional research results to the SFTS field (Xia et al., 2022). Chungbuk National University (BC = 0.56), the Chinese Center for Disease Control and Prevention (BC = 0.21), the University of Chinese Academy of Sciences (BC = 0.21), and the Chinese Academy of Sciences (BC = 0.20) occupy an essential position in the collaborative network.

**Table 2:** The top 5 institutions in number of publications on SFTS.

Rank	Institution	Publications	Citations	Average citation/publication	Centrality
1	National Institute of Infectious Diseases	80	1,688	23.30	0.15
2	Beijing Inst Microbiol & Epidemiol	66	1,644	19.87	0.14
3	Chinese Academy of Sciences	58	1,386	20.08	0.20
4	Anhui Medical University	84	4,474	53.26	0.03
5	Shandong University	50	1,407	31.20	0.03

### 3.4 Analysis of authors

**Figure 5:**
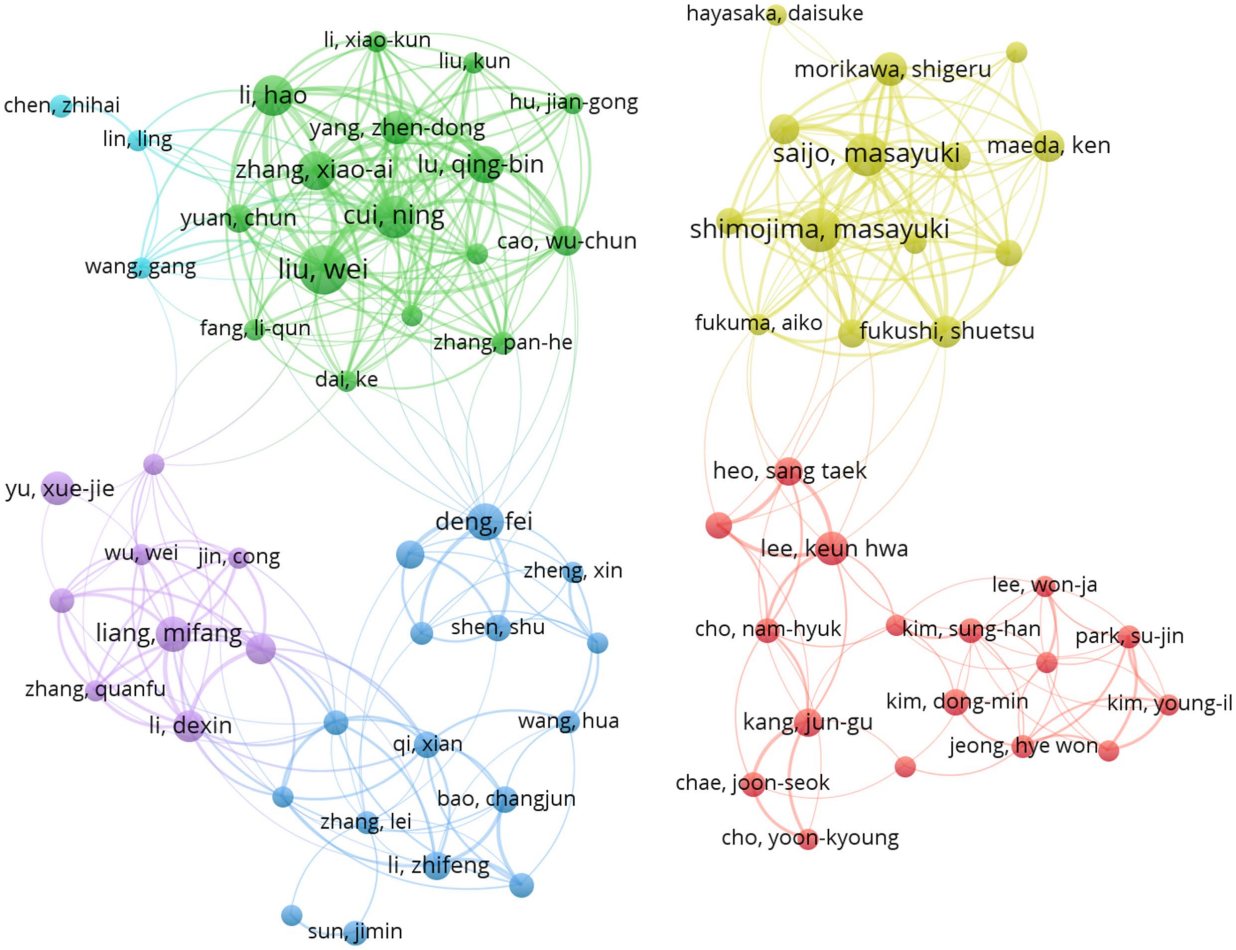
Cooperation map of 75 authors with the number of publications no less than 10 times.

The top three authors in terms of the number of published articles were Liu Wei (*n* = 56), Shimojima Masyuki (*n* = 45), and SaijoMasayuki (*n* = 42) (Table 3). After researchers who had published more than 10 articles related to SFTS were included in the collaborative author network graph using CiteSpace software, the graph consisted of 75 nodes and 380 links (Figure 5). The author collaboration graph reveals the most prolific authors or co-authors while demonstrating their solid collaborative relationship. This tool can provide researchers with information about influential research groups and potential partners, thus helping them to build closer collaboration networks (Xia et al., 2022). From Figure 5, we can see that the 75 authors are divided into 6 different colored clusters. The connecting lines between different clusters are very thin, or there is no connecting line between them, indicating that there is little or no cooperation between the clusters so that the researchers who have profound attainment in the field can work together to produce more high-quality publications and scientific research results through more cooperation and communication.

**Table 3:** The top 5 authors in SFTS field.

Rank	Author	Documents	Citations	Countries/regions	Average citation/publication
1	Liu Wei	56	1,345	China	24.02
2	Shimojima Masyuki	45	1,091	Japan	24.24
3	Saijo Masayuki	42	1,081	Japan	25.74
4	Cui Ning	40	1,361	China	34.03
5	Li Hao	37	922	China	24.92

### 3.5 Analysis of references

**Figure 6:**
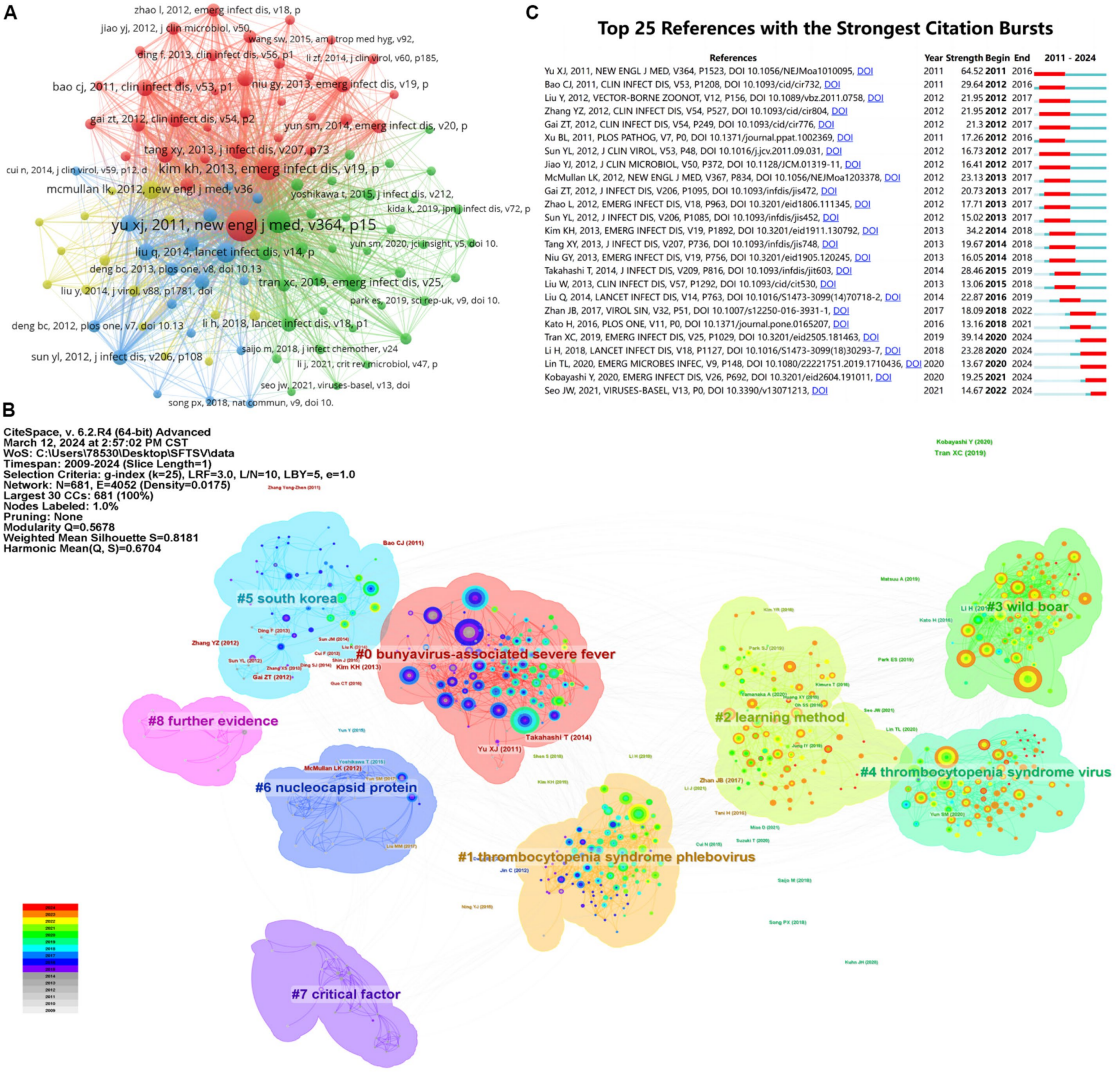
Visualization and analysis of the references in SFTS research. **(A)** Distribution of 104 references with a frequency of no less than 30 times. **(B)** References co-citation clustering network. **(C)** Top 25 references with the strongest citation bursts.

The 760 articles included in this study cited a total of 9,278 references. The minimum number of references was set to 30 using the co-cited literature analysis function of VOSviewer, and 104 articles reached the threshold. As shown in Table 4, the article by Yu et al. (2011) has the highest number of co-citations, which fully proves that this article has significant research value and influence in the field of SFTS. Figure 6A illustrates a reference network containing three clusters. Yu, Takahashi, and Liu Q ranked first in their respective clusters for total co-citations in the blue, red, and green clusters. Nine major sub-themes related to SFTS were identified using CiteSpace’s reference clustering function. The co-citation network analysis of references is shown in Figure 6B, where different colors, from gray to red, represent the number of co-citations in various years. According to the results of the analysis, the Modularity Q is 0.5678 (>0.3) and the weighted mean Silhouette S is 0.8181 (>0.7), both of which are considered to be very high. It indicates a significant clustering structure and convincing clustering results regarding co-citation clustering. The Modularity Q and the weighted mean Silhouette S show excellent clustering effects and network homogeneity properties. The top 25 references with the most robust citation bursts are shown in Figure 6C. The article “Fever with thrombocytopenia associated with a novel bunyavirus in China” published in 2011 is the strongest citation burst with an intensity of 64.53, reflecting this paper’s importance in the SFTS field.

**Table 4:** The top 10 references in SFTS field.

Rank	Author	Co-citation	Year	Journals	DOI
1	Yu XJ	655	2011	New Engl J Med	10.1056/NEJMoa1010095
2	Takahashi T	328	2014	J Infect Dis	10.1093/infdis/jit603
3	Kim KH	318	2013	Emerg Infect Dis	10.3201/eid1911.130792
4	Liu Q	213	2014	Lancet Infect Dis	10.1016/S1473-3099(14)70718-2
5	Mcmullan LK	176	2012	New Engl J Med	10.1056/NEJMoa1203378
6	Tran XC	164	2019	Emerg Infect Dis	10.3201/edi2505.181463
7	Tang XY	163	2013	J Infect Dis	10.1093/infdis/jis748
8	Gai ZT	154	2012	J Infect Dis	10.1093/cid/cir776
9	Bao CJ	151	2011	Clin Infect Dis	10.1093/cid/cir732
10	Zhang YZ	143	2012	Clin Infect Dis	10.1093/cid/cir804

### 3.6 Analysis of journals

**Figure 7:**
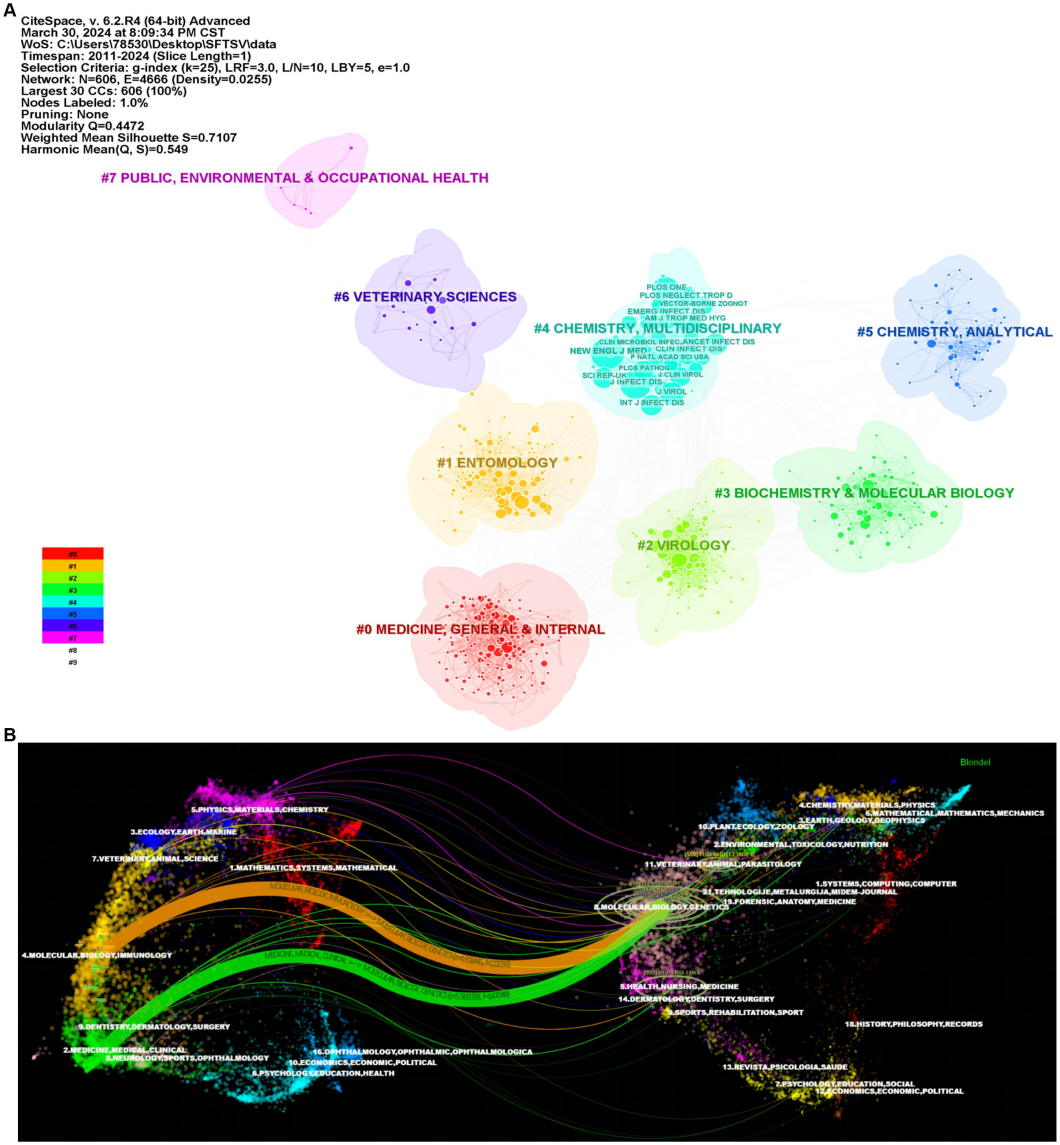
Visualization and analysis of the top journals in SFTS research. **(A)** Subject categories clustering network. **(B)** Dual-map overlap of journals on SFTSV research.

The 760 articles included in this study were published in 188 journals, of which 20 journals published at least 10 articles. Table 5 shows that among the top 10 published journals in SFTS, the top 3 journals were Viruses-Basel (IF = 4.7), PLoS Neglected Tropical Diseases (IF = 3.8), and Emerging Infectious Diseases (IF = 11.8) which published 40, 37, and 35 articles, respectively. Emerging Infectious Diseases (IF = 11.8), Journal of Virology (IF = 5.4), and Journal of infectious Diseases (IF = 6.4) were the top 3 cited journals with 1,679, 1,226 and 979 articles, respectively. In Figure 7A, the eight main areas associated with SFTS-published journals were identified using the journal clustering function of CiteSpace are #Medicine, General & Internal, #Entomology, #Virology, #Biochemistry & Molecular Biology, #Chemistry, Multidisciplinary, #Chemistry, Analytical, #Veterinary Sciences and #Public, Environmental & Occupational Healthy. Figure 7B is a dual-map of the journals on SFTS research by CiteSpace. The cluster positioned on the left signifies the group of journals engaging in citations, whereas the cluster on the right embodies the collection of journals being cited. The dual map shows that there were two paths for citing and cited journals: (1) Molecular, Biology and Immunology-Molecular, Biology, Genetics; (2) Medicine, Medical, Clinical-Molecular, Biology, Genetics. The cited journals are concentrated in 4 circles: (1) Chemistry, Materials, Physics; (2) Veterinary, Animal, Parasitology; (3) Molecular, Biology, Genetics; (4) Health, Nursing, Medicine.

**Table 5:** The top 10 journals in number of publications and citations in SFTS field.

Rank	Publication journal	Documents	Citations	IF^*^	Cited journal	Co-citations	IF^*^
1	Viruses-Basel	40	182	4.7	Emerging Infectious Diseases	1,679	11.8
2	PLoS Neglected Tropical Diseases	37	673	3.8	Journal of Virology	1,226	5.4
3	Emerging Infectious Diseases	35	2,118	11.8	Journal of Infectious Diseases	979	6.4
4	Ticks and Tick-Borne Diseases	28	431	3.2	New England Journal of Medicine	939	158.5
5	Bmc Infectious Diseases	26	203	3.7	Clinical Infectious Diseases	846	11.8
6	International Journal Of Infectious Diseases	25	439	8.4	PLoS ONE	658	3.7
7	American Journal of Tropical Medicine and Hygiene	23	441	3.3	PLoS Neglected Tropical Diseases	553	3.8
8	Journal of Virology	23	1,104	5.4	Proceedings of the National Academy of Sciences of the United States of America	390	11.1
9	PLoS ONE	22	641	3.7	Scientific Reports	388	4.6
10	Scientific Reports	22	476	4.6	American Journal of Tropical Medicine and Hygiene	387	3.3

### 3.7 Analysis of keywords in SFTS research

**Figure 8:**
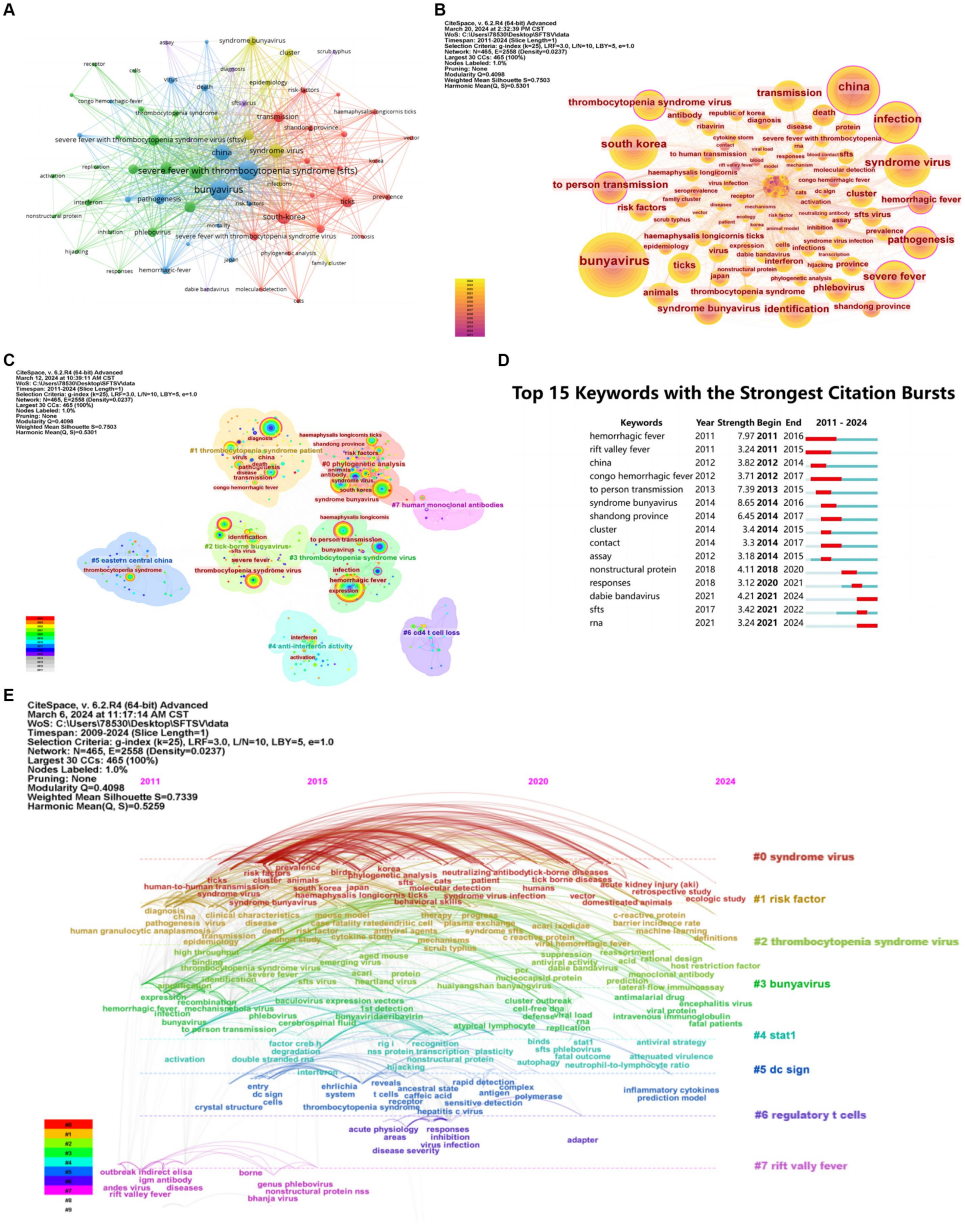
Visualization and analysis of the keywords in SFTS research. **(A)** Distribution of 68 keywords with a frequency of no less than 12 times. **(B)** Co-occurrence of keywords. **(C)** Keywords clustering network. **(D)** Timeline view of keyword cluster. **(E)** Top 15 keywords with the strongest citation bursts.

Because keywords condense the core and essence of a research paper, research hotspots in SFTS-related fields can be identified through keyword co-occurrence analysis. The most common keyword was bunyavirus (Table 6). The keywords with the highest centrality scores were hemorrhagic fever and pathogenesis, and other keywords with high centrality included thrombocytopenia syndrome virus, infection, and severe fever (Table 7). Using VOSviewer to draw keyword co-occurrence graphs for 760 documents, 68 keywords with a frequency greater than or equal to 12 were selected for visualization. The results are shown in Figure 8A. The larger the round nodes are, the more the keywords appear, and the more they represent the hotspots in the field. The node connecting lines represent the strength of association, and the node colors represent the different research topics. The figure’s keyword co-occurrence network diagram with five different color clusters shows the main directions of SFTS research. Figure 8B shows the keyword co-occurrence network graph produced by CiteSpace, where the node size represents how often the keyword occurs, and the color of the year wheel from dark to light indicates how often the keyword occurs from 2011 to 2024. “Bunyavirus,” “China,” and “infection” are the focus of the study. After using the keyword clustering analysis of CiteSpace, eight clusters were obtained, with a Q-value of 0.4098 and an S-value of 0.7503, which shows that the cluster structure is significant and the clustering results are credible (Figure 8C). In the timeline visualization (Figure 8D), the 28 clustered keywords are depicted along the horizontal timeline, showing the research progress in the field of SFTS from 2009 to 2024 as well as the different viruses, risk factors, diagnostic methods, aetiologies, transmission routes, and potential relationships among them. A citation explosion is a sudden surge or a sharp rise in citation frequency in a short period and is an essential reflection of research hotspots over time. The top 15 keywords with the most substantial citation explosion are shown in Figure 8E. The citation explosion in SFTS-related fields started in 2011, among which “hemorrhagic fever” has the most substantial citation explosion, with 7.98. Among them, the keywords “Dabie bandavirus” and “RNA” will continue to be cited in 2024, indicating that they are still hot topics in current research. SFTSV has recently been officially renamed “Dabie bandavirus,” as the study of SFTS progressed, the multifrequency keywords changed. The research focus has gradually shifted from clinical symptoms, mode of transmission, and location of SFTS to the molecular level. The efficacy of various inhibitors has been investigated by studying SFTSV RNA transcription, protein synthesis, and the number of progeny viral particles produced at the molecular level. The natural mutation of SFTSV RNA polymerase enhances viral replication and virulence *in vivo*, providing profound insights into the future prevention and treatment strategies of SFTS and the development of antiviral drugs or vaccines specific to SFTS.

**Table 6:** Top 5 keywords by frequency in SFTS field.

Rank	Keywords	Frequency
1	Bunyavirus	299
2	China	183
3	Syndrome virus	148
4	South Korea	147
5	Infection	135

**Table 7:** Top 5 keywords by centrality in SFTS field.

Rank	Keywords	Centrality
1	Hemorrhagic fever	0.17
2	Pathogenesis	0.15
3	Thrombocytopenia syndrome virus	0.14
4	Infection	0.13
5	Severe fever	0.12

### 3.8 Analysis of timeline of important research on SFTS

**Figure 9:**
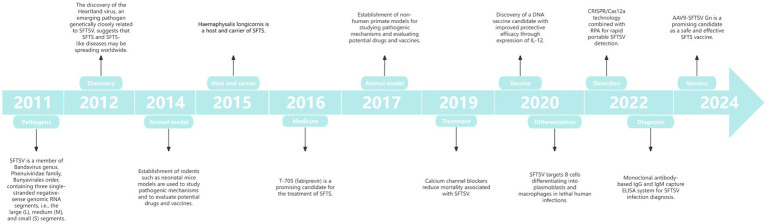
Timeline of important research in the SFTS field.

Research areas are constantly evolving, leading to changes in research hotspots and experimentation. Over the past 15 years, SFTS research has achieved several milestones (Figure 9). Researchers have made significant breakthroughs in studying virus transmission mechanisms and ecology, pathogenesis and pathology, detection and diagnosis, therapeutic methods and vaccine development, and promoting international cooperation and exchange. These studies have deepened our understanding of SFTS and provided an important basis for future prevention, control, and treatment strategies.

## 4 Discussion

SFTS is an emerging tick-borne, highly morbid and fatal disease caused by DBV/SFTS from the *Phenuiviridae* family of the order Bunyavirales, which was first detected in 2009 in central China (Yu et al., 2011). Since the first discovery of SFTSV, the virus has also been widely identified in East Asia (Korea, Japan) and Southeast Asia (Vietnam), and its vector ticks have spread to more than 20 states in the United States (Kim et al., 2024). Therefore, this virus has the potential to cause a large-scale epidemic and has become an urgent global public health crisis. Despite the high morbidity and mortality rates of SFTS, it has not yet been possible to develop a vaccine or an effective antiviral therapy against the disease. With the feverishness of this research field in recent years, the number of studies has steadily increased yearly (Figure 2), and the increasing collaboration between authors and institutions in different countries, relevant bibliometric studies and quantitative analysis of the literature have become particularly important. In this study, 760 relevant research articles from 2009-01-01 to 2024-02-20 were visualized and analyzed using CiteSpace and VOSviewer.

A measure of whether a field is a research hotspot and the period in which it has been a research hotspot lies in the number of papers published. Before 2013, research in the field of SFTS was in its infancy. However, since 2014, the annual number of papers published in the field has continuously increased. The field of SFTS has been a high-profile research hotspot in the last decade, attracting many researchers to explore it in depth and demonstrating intense research interest in this field. China was the first country in the world to publish a report on SFTS with abundant research results. Chinese research institutions are far ahead of the rest of the world in the field of SFTS. As seen in Table 2, the top five institutions in terms of the number of publications are all from China. Most of the collaborations took place among various Chinese institutions, such as the National Institute of Infectious Diseases (NIID), Beijing Institute of Microbiology and Epidemiology (BIMES), Chinese Academy of Sciences (CAS), Anhui Medical University, and Shandong University, which demonstrated China’s profound research strength and close research collaboration network in the field of SFTS. The number of publications, centrality and total citations are three indicators that measure how much a country contributes to a particular field of study [25]. In addition to China, South Korea and Japan also rank high in the number of publications. The United States has the highest number of citations per article. However, the number of articles is lower than that of the previous countries, indicating that the country has many high-quality articles and mature research results. Among the top 5 countries with the most prominent contributions in the field of SFTS, China and the US have the highest TLS values, representing the most significant performance in international collaborations. Most collaborative clusters in Figure 4A are confined within a single country, so more international cooperation is still needed. Close international cooperation and consensus must be further promoted to achieve a surge in research results.

In their 2011 article “Fever with Thrombocytopenia Associated with a Novel Bunyavirus in China,” Yu et al. (2011) described the identification of SFTSV by RNA sequence analysis and electron microscopy. This article was the first to systematically describe the isolation and diagnosis of SFTSV, with the highest citation intensity and number of citations, making it the most important scientific achievement in SFTS research. In recent years, the article with the highest citation intensity is “Endemic Severe Fever with Thrombocytopenia Syndrome, Vietnam,” published in 2019 (Tran et al., 2019). This article deepens our understanding of the distribution of SFTSV in Southeast Asia, revealing that the global distribution of SFTSV may be much larger than previously expected. Further epidemiologic and clinical studies of SFTSV are still needed. This article is also of great significance for the exploration of SFTSV (Tran et al., 2019).

The keyword clustering analysis depicted in Figure 8A offers insights into the prevalent topics and central themes within the SFTS research field. In virology, autophagy assumes a critical role in host defense against various infections and contributes to cellular homeostasis. SFTSV infection can activate autophagy, inducing a complete autophagic flux. Conversely, autophagy facilitates SFTSV infection and replication, supporting the activity of SFTSV ribonucleoproteins involved in transcription and replication processes. Studies focusing on immunological aspects have predominantly examined the antagonistic role of SFTSV nonstructural proteins (NS) in inhibiting RIG-I-like receptor (RLR)-mediated type I interferon (IFN) induction and type I IFN-mediated signaling pathways. The potential interactions between SFTSV and other conserved innate immune responses remain an enigma (Feng et al., 2023). SFTSV gains entry into host cells through glycoprotein internalization, triggering a host immune response against the virus. However, SFTSV has evolved multiple strategies to manipulate host factors, while host genetic factors also play a significant role. Therefore, comprehensive studies on accurately identifying SFTSV and its genetic determinants and elucidating their interactions are imperative for a deeper understanding of SFTSV pathogenesis (Wang et al., 2022).

Numerous researchers have endeavored to establish animal models, recognizing their pivotal role in elucidating the progression of viral diseases, assessing potential therapies, and advancing vaccine and therapeutic development (Clarke and Bradfute, 2020). SFTSV exhibits infectivity across a diverse array of animal species, emphasizing the importance of developing animal models that closely mimic human disease progression (Liu et al., 2014; Kirino et al., 2022). Previous investigations have explored various animal models including non-human primate models, innate immunodeficient mouse models, hamster models, immunocompetent ferret models, and cat models (Casel et al., 2021; Yoshikawa, 2021).

In molecular detection and other studies, researchers have deeply explored the molecular evolutionary trajectory and genetic remodeling process of SFTSVs with the help of complete genome sequences (Lee et al., 2023). They collected complete genome sequence data of SFTSVs isolated globally as of 2019 and conducted a comprehensive analysis of the evolutionary features of SFTSVs. The results show that monitoring SFTSV at the molecular level in both human and non-human hosts is crucial, which helps us gain a deeper understanding of its transmission mechanisms and evolutionary dynamics (Lee et al., 2023). Real-time fluorescence quantitative reverse transcriptase (RT) PCR for detecting viral RNA in serum during the first week of illness has become a compassionate and specific diagnostic tool for diagnosing SFTS in the laboratory. The technique effectively detects viral RNA in serum during the acute infection period and up to 20 days after the onset of symptoms [8]. The period of 7 to 13 days after the onset of the disease is considered critical for the progression of SFTS, and if the serum viral load remains high during this period, it may signal further deterioration of the disease condition or even lead to death. The main risk factors for death in SFTS patients include elevated serum levels of aspartate aminotransferase, lactate dehydrogenase, creatine kinase and their components, and the development of central nervous system symptoms, hemorrhagic manifestations, disseminated intravascular coagulation, and multiorgan failure. In contrast, all clinical markers in surviving patients returned to normal levels during the recovery period (Gai et al., 2012).

Although there are reports that ribavirin may have some impact on patients with illnesses caused by Bunyaviruses, most studies have not found ribavirin to have a significant effect in lowering viral loads or in promoting recovery from thrombocytopenia. Therefore, there are more established and effective treatment options (Liu et al., 2013; Takayama-Ito and Saijo, 2020; Zhang et al., 2021). Moreover, favipiravir (T-705), a pyrazine derivative antiviral drug developed in Japan, has demonstrated stronger antiviral effects than ribavirin in both *in vitro* and *in vivo* trials (Takayama-Ito and Saijo, 2020). Bortezomib, as the first clinically approved proteasome inhibitor, works by regulating the IFN system and apoptotic pathway, thus effectively inhibiting the replication process of SFTSV (Liu et al., 2019). Tilolone has an excellent preventive effect on SFTSV infection and can effectively inhibit SFTSV infection by activating the natural immune response in the *in vitro* and *in vivo* environment (Yang et al., 2022). In vaccine development efforts, live attenuated vaccines, DNA vaccines, fully inactivated viral vaccines, viral vector vaccines, protein subunit vaccines, and mRNA vaccines are being invested in state-of-the-art research in the quest to develop a safe and effective vaccine for SFTSV (Kim et al., 2024). In addition to this, there have been some advances in targeted therapeutic strategies for SFTS (Zhang et al., 2022). SFTSV infection causes abnormal arginine metabolism, leading to decreased platelet counts and T-lymphocyte dysfunction, and decreased platelet nitric oxide (Plt-NO) levels, leading to platelet overactivation and apoptosis. Arginine supplementation can help patients recover faster, which provides new ideas for the unique treatment of SFTS (Li et al., 2018). To date, human-neutralizing monoclonal antibodies (mAb) against SFTSV have been shown to have great potential. They have shown therapeutic efficacy in SFTSV-infected mouse models, and they are expected to be developed into effective antiviral drugs (Wu et al., 2017; Kim et al., 2019; Wu et al., 2020).

From 2021 to the present, “Dabie bandavirus” and “RNA” have been the keywords with high frequency, which means that they are the focus of research in recent years. DBV is a novel negative-stranded RNA ribbon virus in the *Phenuiviridae* family with three genome segments: large, medium and small (Kim and Park, 2023). In Fu et al.’s (2016) study, strains were categorized into six genotypes A-F by phylogenetic analysis, which is the most widely used method of DBV genotyping today, and genotype B can be further categorized into subgenotypes B1, B2, and B3 (Liu et al., 2023). Different clinical symptoms and mortality rates in SFTS patients were associated with infection with different DBV genotypes. In Japan and Korea, the most prevalent genotype was subtype B-2, with mortality rates of 35 and 23.3% for SFTS, respectively (Yokomizo et al., 2022). Genotypes A, D and F are predominant in China, with a low mortality rate of 6.18%.Additional relevant pathology studies have indicated that IL-10 is a relevant immune potential target for treating SFTSV. That inhibition of IL-10 signaling using monoclonal antibodies specifically directed against the IL-10 receptor offers a promising therapeutic avenue for treating patients with lethal SFTS (Kang et al., 2023). Innate immune evasion and induction of pro-inflammatory responses may play dual roles in the pathogenesis of SFTSV, in which the viral NSs proteins utilize IFN antagonism to successfully evade the host’s innate immune defenses, leading to productive viral replication. In order to gain insight into the mechanisms of disease progression in patients infected with SFTSV, further studies are urgently needed to clarify the changes in cytokine dynamics over time. This research is key to unlocking new strategies for disease treatment (Mendoza et al., 2019). Overall, many issues still need to be resolved in SFTS research.

Visual analytical analysis of bibliometrics can help us review and summarize research results, provide insights, and predict future research frontiers and issues. However, the study using CiteSpace and VOSviewer for bibliometric analysis still has some limitations. First, the data we retrieved were limited to English publications in the WOSCC database. Although it is highly respected as a high-quality bibliographic resource database with some authority, some of the documents were not included in the database, making the citation counts underestimated, which would cause a slight bias in our study. Second, this study spans an extensive period of time and is based primarily on quantifiable indicators such as the number of citations and articles, which may not fully reflect the study’s quality, impact, or innovativeness. Third, bibliometric methods mainly analyze the external characteristics of the literature and do not directly assess the quality or innovativeness of the research content. Finally, regarding the use of citation data, the most recent year’s data is unstable and dynamic, changing over time, which can affect the robustness of the analysis to some extent. With the continuous advancement of technology and medicine, the visual analysis of bibliometrics will continue to improve in terms of accuracy and provide more reliable support for academic research. The research hotspots and directions of development in the field of SFTS that it presents can provide researchers with more research ideas.

Bibliometric analysis and data visualization have enabled us to review and summarize research findings in the field of SFTS over the past 15 years and forecast future research frontiers and potential challenges. Presently, academic interest in SFTS has been steadily increasing, with a notable uptick in the number of related publications. China emerges as the foremost contributor to SFTS research, underscoring the importance of fostering international cooperation and knowledge exchange to generate high-quality research outcomes. Through the evolution of high-frequency keywords and the trend of the direction of publications in recent years, the future research of SFTS is developing in the direction of new molecular therapeutics, and the development of related vaccines and drugs is also an essential direction in the future. There are still some gaps and opportunities for research on SFTS. By strengthening research in molecular biology, epidemiology, clinical, and vaccine development, we can gain a deeper understanding of the pathogenesis, transmission pathways, clinical manifestations, etc. of SFTS. Future research on SFTS will center on the prevalence and transmission of the virus, viral characteristics, preventive and control measures, pathogenesis, and new therapeutic methods. Strengthening international cooperation and communication to jointly address global public health challenges is an essential direction for the future.

## Data availability statement

The original contributions presented in the study are included in the article/supplementary material, further inquiries can be directed to the corresponding author.

## Author contributions

HZ: Data curation, Formal analysis, Investigation, Writing – original draft. LZ: Conceptualization, Data curation, Writing – review & editing.
