# Supplementary figures and images for: Knowledge mapping of severe fever with thrombocytopenia syndrome: a bibliometric analysis
Source: Front Microbiol. 2024 Jul 30;15:1423181. doi: 10.3389/fmicb.2024.1423181 (PMC11319145; doi:10.3389/fmicb.2024.1423181)

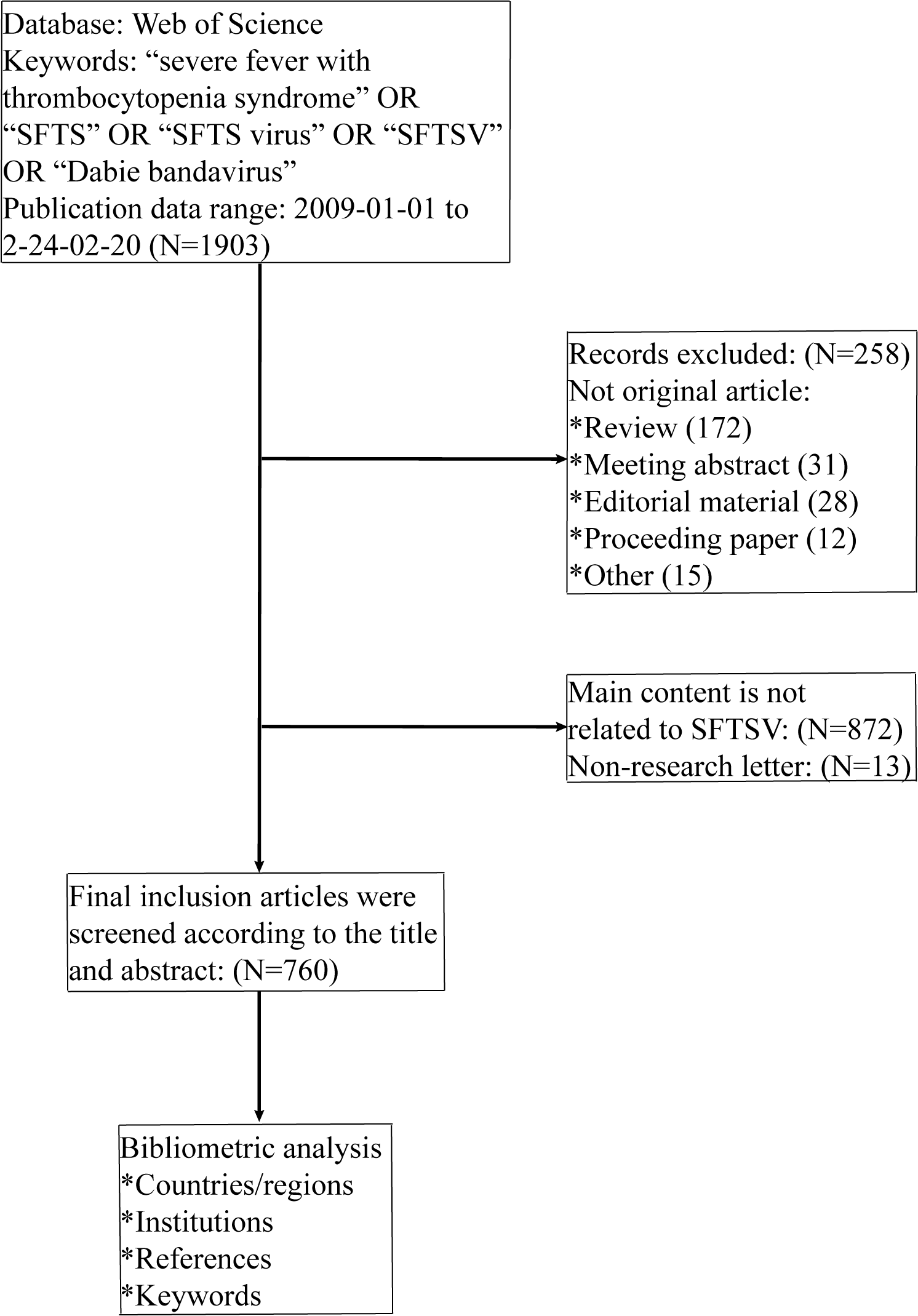

Supplement: Supplementary file 1 [file Figure_1.tif]
